# Reconfigurable Filtering of Neuro-Spike Communications Using Synthetically Engineered Logic Circuits

**DOI:** 10.3389/fncom.2020.556628

**Published:** 2020-10-15

**Authors:** Geoflly L. Adonias, Harun Siljak, Michael Taynnan Barros, Nicola Marchetti, Mark White, Sasitharan Balasubramaniam

**Affiliations:** ^1^Telecommunications Software & Systems Group, Waterford Institute of Technology, Waterford, Ireland; ^2^CONNECT Centre, Trinity College Dublin, Dublin, Ireland; ^3^CBIG at Biomeditech, Faculty of Medicine and Health Technology, Tampere University, Tampere, Finland; ^4^School of Computer Science and Electronic Engineering, University of Essex, Colchester, United Kingdom; ^5^Research, Innovation & Graduate Studies, Waterford Institute of Technology, Waterford, Ireland

**Keywords:** neuron, Hodgkin-Huxley, linear model, transfer function, systems theory, epilepsy, filter

## Abstract

High-frequency firing activity can be induced either naturally in a healthy brain as a result of the processing of sensory stimuli or as an uncontrolled synchronous activity characterizing epileptic seizures. As part of this work, we investigate how logic circuits that are engineered in neurons can be used to design spike filters, attenuating high-frequency activity in a neuronal network that can be used to minimize the effects of neurodegenerative disorders such as epilepsy. We propose a reconfigurable filter design built from small neuronal networks that behave as digital logic circuits. We developed a mathematical framework to obtain a transfer function derived from a linearization process of the Hodgkin-Huxley model. Our results suggest that individual gates working as the output of the logic circuits can be used as a reconfigurable filtering technique. Also, as part of the analysis, the analytical model showed similar levels of attenuation in the frequency domain when compared to computational simulations by fine-tuning the synaptic weight. The proposed approach can potentially lead to precise and tunable treatments for neurological conditions that are inspired by communication theory.

## 1. Introduction

Seizure dynamics with either spontaneous and recurrent profiles can occur even in healthy patients during the processing of sensory stimuli or it could manifest itself as an uncontrolled synchronous neural activity in large areas of the brain (Jirsa et al., 2014). Any disruption to the mechanisms that inhibit action potential initiation or the stimulation of processes that facilitate membrane excitation, can prompt seizures. Tackling this disease efficiently is an existing clinical issue where new approaches are constantly being investigated in order to provide precise and reliable strategies in inhibiting or disrupting seizure-triggering populations of neurons. For example, controlling neuron firing threshold can most likely prevent seizure activity, which can often be achieved at a single neuron level (Scharfman, 2007).

The development of techniques for the treatment of this type of neurodegenerative disorder is challenging not only due to the complexity of the brain function and structure but also as a result of the invasiveness and discomfort caused by today's most common neurostimulation or surgery approaches (Rolston et al., 2012). However, due to the lack of success in non-invasive approaches, the immediate future epilepsy treatment will still see invasive methods. This approach must achieve population-level control with state-of-the-art technology in not only neuroengineering but must also integrate other disciplines. Recent advancements in nanotechnology, for instance, have been enabling the development of novel devices at the nano-scale that are capable of improving bio-compatibility. Nanotechnology-based treatment also includes advantages in the treatment precision, patient comfort as well as longer treatment lifetime. However, there still remain numerous challenges in the use of nanotechnology. For example, the passage of chemicals through the blood-brain barrier (BBB) is among the many challenges that disrupt the efficiency of nanoparticles-mediated drug delivery functioning. Challenges still remain as to how nanoparticles that pass through the BBB will diffuse toward specific neural populations. However, if the drug-loaded nanoparticles can be delivered at sufficient concentrations and accurately to a specific location, this can influence neural activities (Bennewitz and Saltzman, 2009; Veletić et al., 2019). As an example, drug delivery targets specific neurodegeneration promoting factors (Feng et al., 2019) by performing a drug-induced control over intracellular, extracellular and synaptic properties that regulate spiking activity (Blier and De Montigny, 1987).

Previous studies on the firing response of neurons have investigated the filtering capabilities either due to realistic synaptic dynamics (Brunel et al., 2001; Moreno-Bote and Parga, 2004) or by naturally manipulating the resting potential of voltage-dependent active conductances of a neuron enhancing its temporal filtering properties (Fortune and Rose, 1997; Motanis et al., 2018). On the other hand, existing analyses do not account for the many molecular control mechanisms that may influence the synaptic activity, e.g., drug. In the case of seizures, the understanding of the drug-induced firing response may allow further analysis on the impact of high-frequency firing on the neural tissue as well as how to desynchronize or slow it down. Frequency-domain analysis has been performed on top of linear models of the Hodgkin-Huxley (HH) formalism to investigate not only the transmission of information through the use of subthreshold electrical stimulation (Khodaei and Pierobon, 2016) but also the influence of axonal demyelination on the propagation of action potentials (Chaubey and Goodwin, 2016). Although Hodgkin-Huxley is not the only neuron model available in the literature, it is one of the most plausible models for computational neuroscience (Long and Fang, 2010). Other proposed models are, for example, integrate-and-fire, Izhikevich and Fitzhugh-Nagumo models (Mishra and Majhi, 2019).

The manipulation of cellular activity, such as neuronal spiking activity, using molecules complexes to mimic logic gates and transistors has also been proposed in the literature. One example is the work of Vogels and Abbott (2005), in which the propagation of neuronal signals in networks of integrate-and-fire models of neurons was investigated and they found that different types of logic gates may arise within the network by either strengthening or weakening specific synapses. Goldental et al. (2014) used identical neurons to propose dynamic logic gates that work based on their historical activities, interconnection profiles, as well as the frequency of stimulation at their input terminals. In our previous works (Adonias et al., 2019; Adonias et al., 2020), we developed several logic gates arranged in groups of three heterogeneous models of neurons, with two working as inputs and one as the output, and performed a queueing-theoretical analysis aiming at the study of such a complex network as a single element behaving as the collective of those cells. Irrespective of the tremendous efforts from the scientific community, these works do not provide a framework of reconfigurable circuits that could pave the way for more sophisticated approaches for neuron control. Further investigation of novel neuronal electronic components constructions is needed to develop bio-compatible and reliable solutions that can address defective neuronal networks. While the scientific community has been witnessing remarkable progress in the manipulation and engineering of the behavior of mammalian cells (Lienert et al., 2014), the existing models do not yield analytical expressions that could be used to model drug-induced filtering capabilities of a neuron and, in particular, incorporating computing paradigms. The main focus of this work is to lay the ground-work of analytical models for digital filters that are designed and engineered into neurons, potentially leading to the development of novel epilepsy treatments.

**Figure 1:**
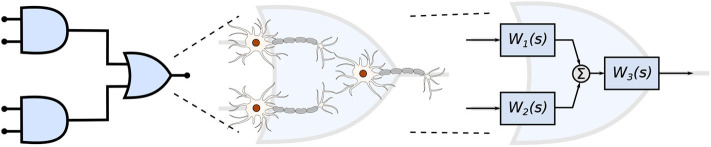
Engineered neuronal digital logic circuit, where each gate is composed of three neurons and each block *W*_*i*_(*s*) represents one neuron as a transfer function to enable communication metric analysis.

In this work, we propose a mathematical framework aiming at the interpretation of the filtering capabilities in small populations of neurons that are engineered into a logic circuit (Figure 1). The circuit aims to reduce the firing rates from its inputs by performing the binary logic as well as integrating reconfigurability, where the different logic circuit arrangements, as well as logic gate types, can be tuned to change the filtering properties. To achieve that in our mathematical framework, we modify parameters on the logic circuit transfer function, derived from the linear interpretation of the Hodgkin-Huxley neuronal model. These parameters are related to neuronal and synaptic properties of a neuro-spike communication, such as conductances and weight, and can potentially be achieved through the sustained administration of a specific drug. Our mathematical framework is, from an application point-of-view, a design platform for neuroscientists in creating filtering solutions for smoothing out the effects of neurological diseases that require the minimization of firing activity. The framework models the effects of drug-induced molecular changes in models of neurons aiming to control the neuronal activity of a synthetic engineered cell, however, the fabrication and specifications of such a drug are out of the scope of this paper. The contributions of this paper are as follows:

- **Neuronal logic circuits are built** using computational models of neurons and this arrangement is expected to be capable of acting as digital filters, converging four inputs into one output with a shift in attenuation driven by modifications to the synaptic weight.
- **A mathematical framework is proposed** paving the way for the design of neuronal digital filters to help suppress the destructive effects of neurodegenerative diseases. This framework should enable the relationship between biophysical models and drug design, facilitating scientists control over the behavior of the filters.
- **Analysis of the performance of the neuronal filters** in terms of accuracy and of signal power attenuated by the circuit. This analysis gives an insight into how parameters such as weight or frequency at the input would affect the performance of such filters.

The remainder of this paper is as follows, section 2.1 briefly describes how neurons differ between each other and how they communicate with one another. In section 2.2, we explain how neurons can function as non-linear electronic circuits based on the seminal work of Hodgkin and Huxley (1952) and we also describe the process of linearization aiming to derive a transfer function of the filter model. The filter design is explained in section 2.3 which also covers how neurons are represented as compartments and connected to form logic gates and, consequently, to form logic circuits. In section 3, we present the results that are discussed in section 4 and, finally, the conclusions are presented in section 5.

## 2. Materials and Methods

### 2.1. Neuronal Communication

To be able to synthetically implement complex functions inside the brain, we must control how the neurons exchange information using the propagation of action potentials inside a network of neurons. The number of excitatory and inhibitory connections between neurons determines the spatio-temporal dynamics of the action potentials propagation (Zhou et al., 2018). Efficient coding and modulation of neuronal information have been used to implement bio-computational approaches in our previous work (Adonias et al., 2020). Bio-computing can be created from neuronal networks that are engineered to function as logic circuits through controlling the neuro-spike communication and curbing the signal propagation dynamics between the neurons.

We aim to investigate the neuronal and synaptic properties in constructing logic circuits that perform the filtering of spikes in small populations from the somatosensory cortex. The cortex is responsible for most of the signal processing performed by the brain and comprises a rich variety of morpho-electrical types of neuronal and non-neuronal cells. We will take into account these characteristics in the construction of our mathematical framework that is used to design the circuits.

#### 2.1.1. Properties of a Neuron

Neurons are divided into three main parts: dendrites, soma, and axon. Dendrites receive stimuli from other cells and the way these dendritic trees are projected onto neighboring neurons in a network helps to classify neuron morphological types. The axon passes stimuli forward to cells connected down the network through its axon terminals and the soma is the main body of the neuron. Each neuron's response to a stimulus will dictate the electrophysiological neuron type. The soma is where most proteins and genes are produced and where stimuli are generated and fired down the axon.

Besides the way dendrites are projected, the proteins and genes that neurons express and their morphological and electrophysiological characteristics are important for the classification of different types of neurons. One of the most comprehensive works on neuronal modeling, by Markram et al. (2015), classifies the neurons from the rat's somatosensory cortex based on their morpho-electrical properties (morphological and electrical characteristics) as well as the cortical layer they belong (columnar and laminar organization).

##### 2.1.1.1. Morpho-electrical characteristics

Even though all neurons used in this work can assume different morphological structure, it is exactly by analyzing their axonal and dendritic ramification that we can have a good enough categorization of their respective morphological types. Regardless of their types, neurons in the cortical layer are considered of small sizes (8 - 16μm). Furthermore, inhibitory neurons can be better identified by their axonal features while excitatory neurons can be more easily classified based on their dendritic features (Markram et al., 2015). Each morphological type (m-type) can fire different spiking patterns and this may affect the gating capabilities of neurons due to the fluctuations on precise spike timing. Markram et al. (2015) categorized 11 different electrical types (e-types) of neurons, hence, 11 different ways of firing a spike train generated in response to an injected step current.

##### 2.1.1.2. Cortical organization

The cerebral cortex comprises six distinguished horizontal layers of neurons, with each layer having particular characteristics such as cell density and type, layer size, and thickness. This horizontal configuration is also known as a “laminar” organization, where the layers are identified as (1) Molecular layer, which contains only a few scattered neurons and consists mostly of glial cells and axonal and dendritic connections of neurons from other layers; (2) External granular layer, containing several stellate and small pyramidal neurons; (3) Pyramidal layer, contains non-pyramidal and pyramidal cells of small and medium sizes; (4) Inner granular layer, predominantly populated with stellate and pyramidal cells, this is the target of thalamic inputs; (5) Ganglionic layer, containing large pyramidal cells that establish connections with subcortical structures; and (6) Multiform layer, populated by just a few large pyramidal neurons and a good amount of multiform neurons, which sends information back to the thalamus. All layers may contain inter-neurons bridging two different brain regions.

The neurons are not just stacked one on top of another suggesting a horizontal organization, indeed vertical connections are also found in between the neurons from either the same or different layers. This allows another type of classification known as mini-columns (also called, micro-columns) with a diameter of 30–50 μm and when activated by peripheral stimuli, they are seen as macro-columns, with a diameter of 0.4–0.5 mm (Peters, 2010). This will create network topologies with intrinsic characteristics, e.g., connection probabilities between neurons, that influence the signal propagation to converge into either a specific pattern or flow.

#### 2.1.2. Neuron-to-Neuron Communication

**Figure 2:**
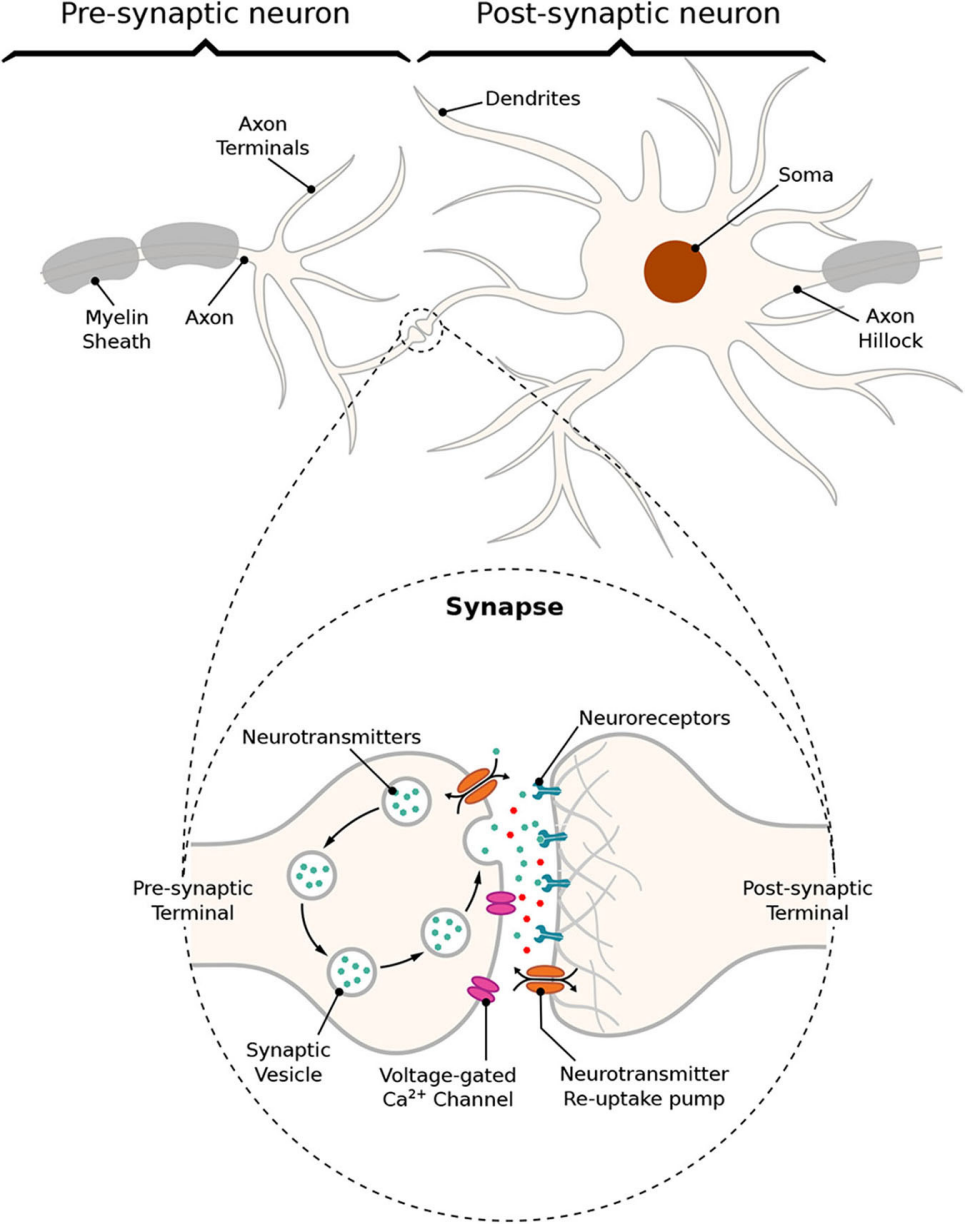
Schematic of a *synapse*; action potentials traveling down the axon trigger the release of neurotransmitters into the cleft between pre- and post-synaptic terminals, traveling toward neuroreceptors on the other end leading to changes on membrane conductance that can either excite or inhibit the post-synaptic neuron.

The communication between a pair of neurons is done through the diffusion of neurotransmitters in the synaptic cleft; this process is triggered by an electrical impulse reaching the axon terminals of the transmitting cell characterizing an electrochemical signaling process known as the *synapse*. Action potentials propagate down the axon of the pre-synaptic cell, which is the sender cell, and when reaching the axon terminals also known as pre-synaptic terminals, it triggers the release of vesicles containing neurotransmitters into the synaptic cleft, which is the gap between a pre- and a post-synaptic terminal, as illustrated in Figure 2. Those neurotransmitters will probabilistically bind to neuro-receptors located at the post-synaptic terminals, i.e., dendrites (Balevi and Akan, 2013), triggering the exchange of ions through the membrane that can either excite or inhibit the cell, depending on the type of neurotransmitters that were received. In our work, we focus on the synaptic weight between the pre- and post-synaptic terminals. The synaptic weight is a measure of how much influence the pre-synaptic stimuli have on the post-synaptic cell and it is known to have its value best approximated to the time integral of the synaptic conductance (Gardner, 1989). Furthermore, the value of synaptic conductance in the post-synaptic terminal is driven by the number of neurotransmitters bound to neuroreceptors (Guillamon et al., 2006). We illustrate the synaptic weight, in Figure 2, as red neurotransmitters which should have their release from the pre-synaptic terminals induced by the administration of a specific drug.

In an excitatory synapse, the membrane potential of the post-synaptic cell, which rests at approximately −65 mV, will start depolarizing itself until it reaches a threshold, *th*, for action potential initiation. On the other hand, if the synapse is inhibitory, the membrane should get even more polarized making it nearly impossible for the cell to fire a spike and not allowing the propagation of any signal down the network from the inhibited cell. After reaching *th*, the membrane potential should increase toward a maximum peak of depolarization, and then the cell will start the process of repolarization toward its resting potential. For a brief moment, the potential inside the cell will cross the level of potential when at rest making the membrane hyperpolarized, which is a period known as the *refractory period* and it can be further subdivided as *absolute* and *relative*. The absolute refractory period (ARP) lasts around 1–2 ms during which the neuron is unable to fire again regardless of the strength of the stimuli; then, it is followed by the relative refractory period (RRP) during which a response in the potential of the cell may be evoked depending on the strength of the stimuli (Mishra and Majhi, 2019).

### 2.2. Electronic Interpretation of a Neuron Model

The main structures of a neuron, previously mentioned in section 2.1.1, can assume different shapes and spatial structures that play an important role in determining its input and output relationship. By sectioning the neuron into several compartment models, we are able to account for the influence that individual compartments have on the communication process of the neuron. Even though we consider the same value of resting potential for all compartments of the cell, there is some discussion on whether different compartments have different potentials when at rest (Hu and Bean, 2018).

We aim to develop a transfer function for the neuron-spike response, or output [*V*(*s*)], to a particular spike input [*I*(*s*)]. Using a transfer function for each neuron which is represented as a single compartment, we are able to efficiently associate the configuration of the filters with the structure of the neural network as well as the individual characteristics of each neuron. On top of that, we also are able to focus on frequency domain for an effective spike firing filtering. We rely on the electronic interpretation of the Hodgkin-Huxley model of neuron action potentials, which is made based on the neuronal cable theory assumptions on the static ionic channels conductance. In this section, we provide the details of the development of the transfer function, which is built on the linearization process of the Hodgkin-Huxley neuron model.

#### 2.2.1. Hodgkin-Huxley Formalism

**Figure 3:**
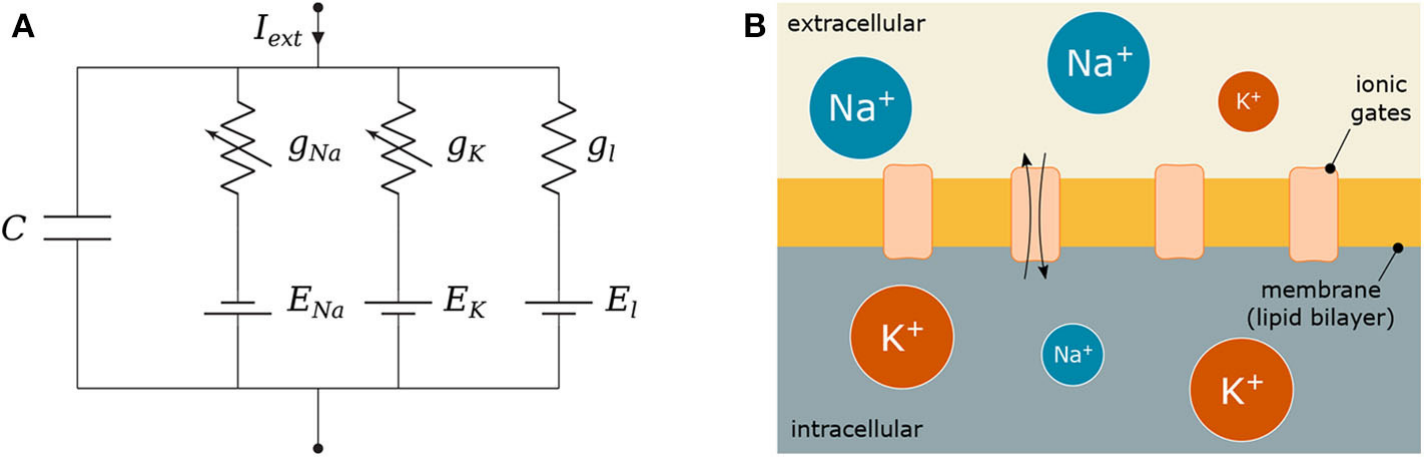
Hodgkin-Huxley (HH) model: **(A)** Electronic circuit representation and **(B)** Equivalent biological HH compartment; the lipid bilayer is modeled as *C*, the conductances *g* represent how open or close the ionic gates are and the gradient of ions between the intra- and extra-cellular space define the reversal potentials *E*.

As aforementioned in section 1, neurons can perform spike filtering tasks either by manipulating ionic conductances, such as sodium and potassium conductances, from within the cell (Fortune and Rose, 1997) or by working on the extracellular environment where the synapse occurs (Brunel et al., 2001; Moreno-Bote and Parga, 2004). Furthermore, filtering capabilities may vary according to the non-linearities of the neuron's activity and action potential propagation. In order to design an efficient filtering process, we will need to eliminate the non-linearities so we can directly link neurons properties to the filtering behavior and adjust these properties according to a desired filtering performance level. We consider the Hodgkin and Huxley non-linear model (Pospischil et al., 2008) as our basic model since it perfectly describes the influence of ionic conductance and synaptic conductance in the propagation of the action potentials. We assume that parts of the neuron will constitute a compartment, which results in the electric circuit in Figure 3A when applying the conventional neural cable theory.

Figure 3 depicts *C* as the membrane capacitance, each voltage-gated ionic channel represented by its respective conductances *g*_*Na*_ and *g*_*K*_ and the leak channel by the linear conductance *g*_*l*_. The membrane capacitance is proportional to the surface area of the neuron and, along with its resistance, dictates how fast its potential responds to the ionic flow. The ratio between intra- and extra-cellular ions define the reversal potentials *E*_*Na, K, l*_ establishing a gradient that will drive the flow of ions (Barreto and Cressman, 2011).

When an external stimulus, *I*_*ext*_, is presented, it triggers either the activation or inactivation of the ionic channels that allow the exchange of ions that result in depolarization (or hyperpolarization when inhibitory) of the membrane of the cell. These dynamics are modeled as

(1) $$\begin{array}{l} C\frac{\text{d}V}{\text{d}t}=-I_{l}-I_{Na}-I_{K}+I_{ext}, \end{array}$$

where *V* is the membrane potential and *I*_*x*_ are the ionic currents where *x* represents either a specific ion (*Na*, *K*) or the leak channel (*l*). Those currents are described as

(2) $$\begin{array}{l} I_{l}=g_{l}\left(V-E_{l}\right), \end{array}$$

(3) $$\begin{array}{l} I_{Na}=g_{Na}m^{3}h\left(V-E_{Na}\right), \end{array}$$

(4) $$\begin{array}{l} I_{K}=g_{K}n^{4}\left(V-E_{K}\right), \end{array}$$

where *m* and *h* are the activation and inactivation variables of the sodium channel, respectively, and *n* is the activation variable of the potassium channel, following the conventional approach described by Hodgkin and Huxley (1952) and stated as

(5) $$\begin{array}{l} \frac{\text{d}m}{\text{d}t}=\alpha_{m}\left(V\right)\left(1-m\right)-\beta_{m}\left(V\right)m, \end{array}$$

(6) $$\begin{array}{l} \frac{\text{d}h}{\text{d}t}=\alpha_{h}\left(V\right)\left(1-h\right)-\beta_{h}\left(V\right)h, \end{array}$$

(7) $$\begin{array}{l} \frac{\text{d}n}{\text{d}t}=\alpha_{n}\left(V\right)\left(1-n\right)-\beta_{n}\left(V\right)n, \end{array}$$

in which the values of the rate constants α_*i*_ and β_*i*_ for the *i*-th ionic channel can be defined as

(8) $$\begin{array}{l} \alpha_{m}=\frac{0.1\left(V+40\right)}{1+e^{-\left(V+40\right)/10}}, \end{array}$$

(9) $$\begin{array}{l} \beta_{m}=4e^{-\left(V+65\right)/20}, \end{array}$$

(10) $$\begin{array}{l} \alpha_{h}=0.07e^{-\left(V+65\right)/20}, \end{array}$$

(11) $$\begin{array}{l} \beta_{h}=\frac{1}{1+e^{-\left(V+35\right)/10}}, \end{array}$$

(12) $$\begin{array}{l} \alpha_{n}=\frac{0.01\left(V+55\right)}{1-e^{-\left(V+55\right)/10}}, \end{array}$$

(13) $$\begin{array}{l} \beta_{n}=0.125e^{-\left(V+65\right)/80}. \end{array}$$

The membrane capacitance is proportional to the size of the cell, and on the other hand, the bigger the cell diameter, the lower the spontaneous firing rate (Sengupta et al., 2013). Furthermore, each ionic channel can be studied as containing one or more physical gates which can assume either a permissive or a non-permissive state when controlling the flow of ions. The channel is open when all gates are in the permissive state, and it is closed when all of them are in the non-permissive state (Baxter and Byrne, 2014).

#### 2.2.2. Hodgkin-Huxley Linear Model

In order to derive a transfer function for the Hodgkin-Huxley model, we must consider each neuron as a system that is linear and time-invariant (LTI). If the system is non-linear, then a linearization process should be done before any frequency analysis is performed. For a more detailed analysis on the procedures for linearization of the Hodgkin-Huxley model, the reader is referred to Koch (2004), Mauro et al. (1970), Sabah and Leibovic (1969), and Chandler et al. (1962).

The linearization process requires that we reconsider the electronic components in each neuron compartment to adequately eliminate trivial relationships. Membranes with specific types of voltage- and time-dependent conductances can behave as if they had inductances even though neurobiology does not possess any coil-like elements. This modification will transform the behavior of non-linear components toward linearization, resulting in a proportional relationship between the voltage and current changes (Koch, 2004).

**Figure 4:**
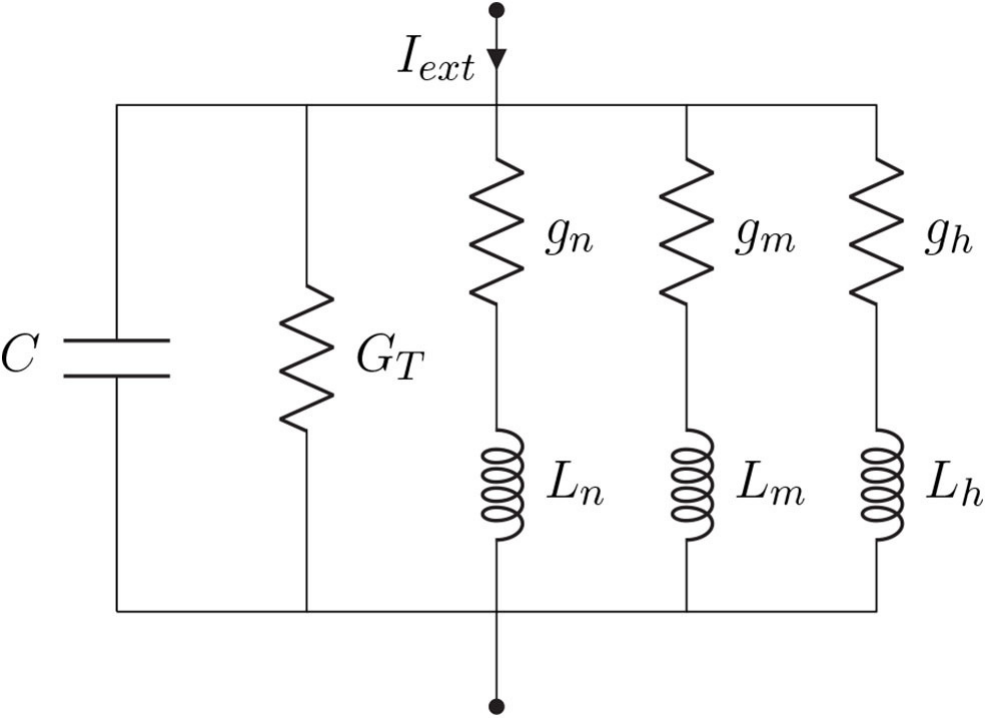
Hodgkin-Huxley linear circuit model representation.

Every linearization process is performed for small variations around a fixed point, hereafter denominated by δ, and in the case of the Hodgkin-Huxley model, this fixed point should be the steady-state (resting state) of the system. Because the sodium activation generates a current component that flows in an opposite direction compared to that of a passive current, the branch concerning the sodium activation should have components with negative values while the branches regarding potassium activation and sodium inactivation should have components with positive values (Sabah and Leibovic, 1969). The linear version of the circuit of Figure 3A is illustrated in Figure 4, where *C* is the membrane capacitance, *g*_*n*_, *g*_*m*_, and *g*_*h*_ are the conductances of the inductive branches connected in series with their respective inductances *L*_*n*_, *L*_*m*_, and *L*_*h*_ derived from the linearization process and *G*_*T*_ = *G*_*L*_ + *G*_*K*_ + *G*_*Na*_ is the total pure membrane conductance.

Let us consider the membrane potential deviation, δ*V*, around some fixed potential. Thus, we can express the response of the circuit to small-signal inputs as

(14) $$\begin{array}{l} C\frac{d\delta V}{dt}=I_{ext}-\delta I_{l}-\delta I_{K}-\delta I_{Na}, \end{array}$$

where δ*I*_*l,Na,K*_ are current variations at any given steady-state and can be defined as

(15) $$\begin{array}{l} \delta I_{l}=g_{l}\delta V, \end{array}$$

(16) $$\begin{array}{l} \delta I_{K}=G_{K}\delta V+4g_{K}n_{\infty }^{3}\left(V-E_{K}\right)\delta n, \end{array}$$

(17) $$\begin{array}{l} \delta I_{Na}=G_{Na}\delta V+3g_{Na}m_{\infty }^{2}h_{\infty }\left(V-E_{Na}\right)\delta m\\ \;+g_{Na}m_{\infty }^{3}\left(V-E_{Na}\right)\delta h, \end{array}$$

where *G*_*K,Na*_ are pure conductances of potassium and sodium and *G*_*L*_ the pure leak conductance expressed as

(18) $$\begin{array}{l} G_{L}={\bar{g}}_{l}, \end{array}$$

(19) $$\begin{array}{l} G_{K}={\bar{g}}_{K}n_{\infty }^{4}, \end{array}$$

(20) $$\begin{array}{l} G_{Na}={\bar{g}}_{Na}m_{\infty }^{3}h_{\infty }, \end{array}$$

where ḡ_*K,Na*_ are the maximum attainable conductances, and δ*n*, δ*m*, and δ*h* are small variations around the steady-state of the activation and inactivation variables *n*, *m*, and *h* which are written as

(21) $$\begin{array}{l} \frac{d\delta n}{dt}=\frac{d\alpha_{n}}{dV}\delta V-\left(\alpha_{n}+\beta_{n}\right)\delta V-n_{\infty }\left(\frac{d\alpha_{n}}{dt}-\frac{d\beta_{n}}{dt}\right)\delta V, \end{array}$$

(22) $$\begin{array}{l} \frac{d\delta m}{dt}=\frac{d\alpha_{m}}{dV}\delta V-\left(\alpha_{m}+\beta_{m}\right)\delta V-m_{\infty }\left(\frac{d\alpha_{m}}{dt}-\frac{d\beta_{m}}{dt}\right)\delta V, \end{array}$$

(23) $$\begin{array}{l} \frac{d\delta h}{dt}=\frac{d\alpha_{h}}{dV}\delta V-\left(\alpha_{h}+\beta_{h}\right)\delta V-h_{\infty }\left(\frac{d\alpha_{h}}{dt}-\frac{d\beta_{h}}{dt}\right)\delta V, \end{array}$$

as a function of the derivative of the rate constants α_*n,m,h*_ and β_*n,m,h*_, and *n*_∞_, *m*_∞_, and *h*_∞_ are the steady-state values of *m*, *n*, and *h* defined as

(24) $$\begin{array}{l} n_{\infty }=\frac{\alpha_{n}}{\alpha_{n}+\beta_{n}}, \end{array}$$

(25) $$\begin{array}{l} m_{\infty }=\frac{\alpha_{m}}{\alpha_{m}+\beta_{m}}, \end{array}$$

(26) $$\begin{array}{l} h_{\infty }=\frac{\alpha_{h}}{\alpha_{h}+\beta_{h}}, \end{array}$$

and the conductances, *g*_*n,m,h*_, and inductances, *L*_*n,m,h*_, of the inductive branches are defined as

(27) $$\begin{array}{l} g_{n}=\frac{4{\bar{g}}_{K}n_{\infty }^{3}\left(V-E_{K}\right)\left[\frac{d\alpha_{n}}{dV}{|}_{r}-n_{\infty }\frac{d\left(\alpha_{n}+\beta_{n}\right)}{dV}{|}_{r}\right]}{\alpha_{n}+\beta_{n}}, \end{array}$$

(28) $$\begin{array}{l} L_{n}=\frac{1}{g_{n}\left(\alpha_{n}+\beta_{n}\right)}, \end{array}$$

(29) $$\begin{array}{l} g_{m}=\frac{3{\bar{g}}_{Na}m_{\infty }^{2}h_{\infty }\left(V-E_{Na}\right)\left[\frac{d\alpha_{m}}{dV}{|}_{r}-m_{\infty }\frac{d\left(\alpha_{m}+\beta_{m}\right)}{dV}{|}_{r}\right]}{\alpha_{m}+\beta_{m}}, \end{array}$$

(30) $$\begin{array}{l} L_{m}=\frac{1}{g_{m}\left(\alpha_{m}+\beta_{m}\right)}, \end{array}$$

(31) $$\begin{array}{l} g_{h}=\frac{{\bar{g}}_{Na}m_{\infty }^{3}\left(V-E_{Na}\right)\left[\frac{d\alpha_{h}}{dV}{|}_{r}-h_{\infty }\frac{d\left(\alpha_{h}+\beta_{h}\right)}{dV}{|}_{r}\right]}{\alpha_{h}+\beta_{h}}, \end{array}$$

(32) $$\begin{array}{l} L_{h}=\frac{1}{g_{h}\left(\alpha_{h}+\beta_{h}\right)}. \end{array}$$

Each channel has a probability of being open which represents the fraction of gates in that channel that are in the permissive state (Gerstner et al., 2014). The gating variables are described by the coupling of the conductances *g*_*n,m,h*_ and their respective inductances *L*_*n,m,h*_ which are functions of the rate constants representing the transition from permissive to non-permissive state, α(*V*), and vice-versa, β(*V*) which should take a short period of time, τ = [α(*V*) + β(*V*)]^−1^, to eventually reach a steady-state value, α_∞_ and β_∞_ (Koslow and Subramaniam, 2005).

Borrowing concepts from systems theory such as frequency analysis of LTI systems, as a standard procedure for the analysis of linear differential equations as simpler algebraic expressions (see Nise, 2015), and the linearization of non-linear systems for the reason previously mentioned at the beginning of this section, we derived a transfer function in the *Laplace* domain for the linear system from Figure 4. The relationship between the different elements of the circuit and their respective impedance and admittance values from the *Laplace* transforms are depicted in Table 1.

**Table 1:** Impedance relationships for capacitors, resistors, and inductors.

**Component**	**Impedance**	**Admittance**
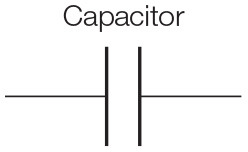	$\frac{1}{Cs}$	*Cs*
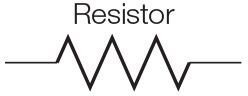	*R*	$G=\frac{1}{R}$
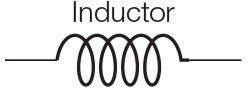	*Ls*	$\frac{1}{Ls}$

Therefore, the relationship between the output and the input of the system in the frequency domain is expressed as

(33) $$\begin{array}{l} \frac{V\left(s\right)}{I\left(s\right)}=\\ \frac{s^{3}L_{n}L_{m}L_{h}}{\left\{L_{n}L_{m}L_{h}\left[s^{4}C+s^{3}\left(G_{T}+g_{n}+g_{m}+g_{h}\right)\right]+s^{2}\left(L_{m}L_{h}+L_{n}L_{h}+L_{m}L_{h}\right)\right\}} \end{array}$$

where *s* = σ + *jω* is a complex variable; $j=\sqrt{-1}$ and ω = 2π*f*, where *f* is the frequency in Hertz. Let us rewrite Equation (33) as

(34) $$\begin{array}{l} W\left(s\right)=C^{-1}\frac{s}{s^{2}+sC^{-1}\left(G_{T}+g_{n}+g_{m}+g_{h}\right)+C^{-1}\left(L_{m}^{-1}+L_{n}^{-1}+L_{h}^{-1}\right)}. \end{array}$$

Now, denoting γ = *G*_*T*_+*g*_*n*_+*g*_*m*_+*g*_*h*_ and $\lambda^{-1}=L_{n}^{-1}+L_{m}^{-1}+L_{h}^{-1}$ and performing a few algebraic manipulations, we end up with the following transfer function for the filter model

(35) $$\begin{array}{l} W\left(s\right)=\gamma^{-1}\frac{C^{-1}\gamma s}{s^{2}+C^{-1}\gamma s+\lambda^{-1}C^{-1}}. \end{array}$$

For frequency response analysis, we observe the behavior of *W*(*jω*), i.e., substitute *s* = *jω*. For ω → 0, *W*(*jω*) behaves like ω; for ω → ∞ it behaves like $\frac{1}{\omega +1}$, i.e., in both cases it tends to zero, and hence demonstrates the behavior of a second-order band-pass filter (BPF). It corresponds to the canonical form $\frac{K\left(\omega_{0}/Q\right)s}{s^{2}+\left(\omega_{0}/Q\right)s+\omega_{0}^{2}}$ where *K* = γ^−1^ is the gain, $Q=\gamma^{-1}\sqrt{C\lambda^{-1}}$ is the selectivity and $\omega_{0}=\sqrt{\lambda^{-1}C^{-1}}$ is the peak frequency of the filter. This agrees with findings from previous literature on the matter (Plesser and Geisel, 1999) that concluded the periodicity of a stimulus is optimally encoded by a neuron only in a specific spectral window.

### 2.3. Transfer Function Filter Design

Given the transfer function for a neural compartment in the previous section, we now progress toward a transfer function for the spike filter. The filter is comprised of neurons that are particularly chosen to have a network that will behave as a digital gate and a small population that will behave as a circuit that implements the filter. Our aim is to capture the relationship between compartments as well as neuron connections so we can build a transfer function for the filter while considering neuron connection variables (synaptic conductance and synaptic weight) that allow easy reconfiguration of the filtering process. The linearization process combined with the analysis of the neuron communications is the driver of the filtering process, which also allows the derivation of a filter transfer function which is detailed below.

#### 2.3.1. Biological Logic Gates and Circuits

Synthetic biology is the technology that allows the control of the neurons' internal process in order to construct non-natural activity and functioning of neurons, e.g., logic gates (Larouche and Aguilar, 2018). Synthetic logic operations inspire scientists to address the challenges posed by novel synthetic biomedical systems, such as biocompatibility and long-term use.

**Figure 5:**
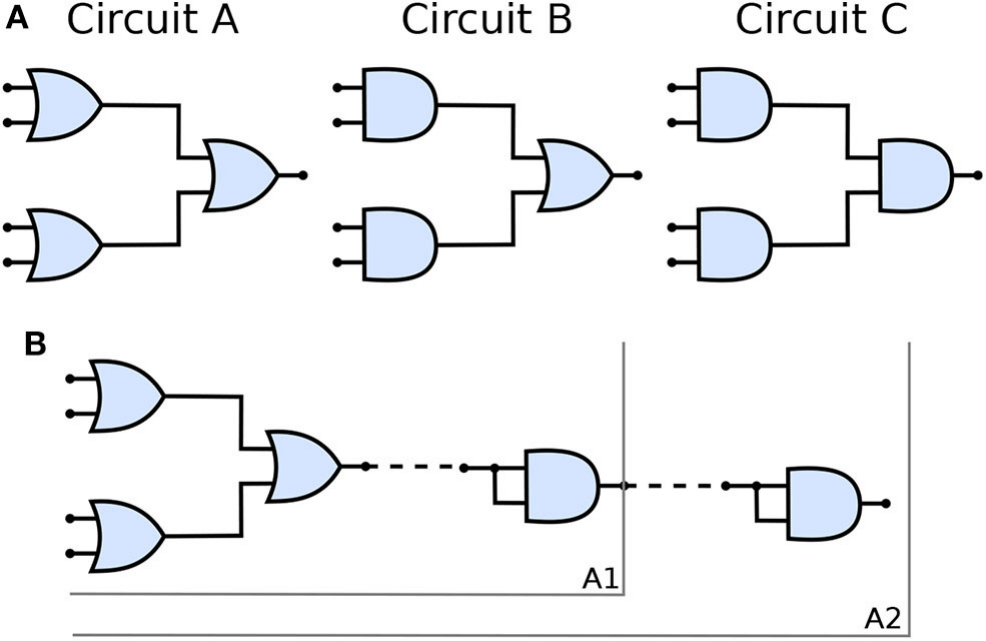
**(A)** Schematic of circuits A, B, and C and **(B)** The connection of AND gates in cascade to circuit A. *A*1 refers to the arrangement described by a single AND gate connected to the output of the circuit A and *A*2 refers to another AND gate connected to the output of *A*1 arrangement, i.e., two AND gates in cascade with circuit A. Analogous nomenclature is employed for both circuits B, as in B1/B2 and C, as in C1/C2.

Figure 5A shows the three types of the circuit we have built and analyzed in this work. From circuits A to C, the number of OR gates is decreased; when compared to AND gates, OR gates are quite permissive. In our previous study (Adonias et al., 2020), we present an analysis on how signals from two input neurons will need to be close to each other to amplify the action potential of the output neuron in order to achieve maximum AND-gating accuracy. The transformation from a purely OR-formed logic circuit to a purely AND-formed one leads to the confirmation of what the truth-tables suggest, i.e., fewer states evoke spikes in the output and, consequently, the attenuation of higher frequencies in the inputs. Figure 5B shows the connection of AND gates in cascade, and this analysis is further discussed in the section 4. Each of the circuits was analyzed with one and two AND's in cascade, hence the nomenclature of a letter followed by a number, the letter refers to the type of circuit and the number accounts for how many AND gates are connected in cascade. Only two types of logic gates were used to build the circuits, an AND composed of the cells L23-MC (Layer 2/3 Martinotti Cell), L23-NBC (Layer 2/3 Nest Basket Cell), and L1-HAC (Layer 1 Horizontal Axon Cell); and an OR composed of the cells L23-MC, L23-NBC, and L1-DAC (Layer 1 Descending Axon Cell). These cells were picked because they showed the best performance in our previous analysis on their individual gating capabilities.

Given that several factors such as connection probability, type of cell, and different numbers of compartments (as discussed in section 2.3.2) among different types of neurons may influence its gating capabilities. This variation on the quantity of compartments could also lead to variations on periods for the action potential to reach the post-synaptic terminals and start the synapse process. Furthermore, cells with bigger sizes of soma may take more time and amount of stimuli to reach threshold for action potential initiation (Sengupta et al., 2013), thus, also affecting the way a neuronal logic gate would work regarding a specific morphological neuronal type. For that reason, it is safe to keep two cells fixed as inputs (as illustrated in Figure 1) and then deploy an arrangement with which its performance has been previously assessed, allowing us to be fairly certain about how the synthetic gate or circuit should behave. Each neuron is represented by a block, *W*_*i*_(*s*) for the *i*-th neuron, and its representation in the frequency domain is proposed in Equation (35) and further detailed in section 2.3.2.

#### 2.3.2. Compartmental Modeling

Neurons are very complex structures with numerous ramifications and several factors that contribute to their highly non-linear dynamism. Aiming to make the comprehension of such a complex electrical behavior easier, one employs a widely used technique called “compartmental modeling.” Since different neurons have different morphologies, the mechanism of determining the number of compartments will be based on estimating the length of a specific neuronal structure. For instance, a varying length of axon, which will reflect in different quantities of compartment in series, where we will have a fixed size for each segment of the axon representing one compartment. This is a very natural and elegant way to model dynamic systems as multiple interconnected compartments where each compartment is described by its own set of equations, carrying the influence of one compartment to the next reproducing the behavior of the whole neuron.

**Figure 6:**
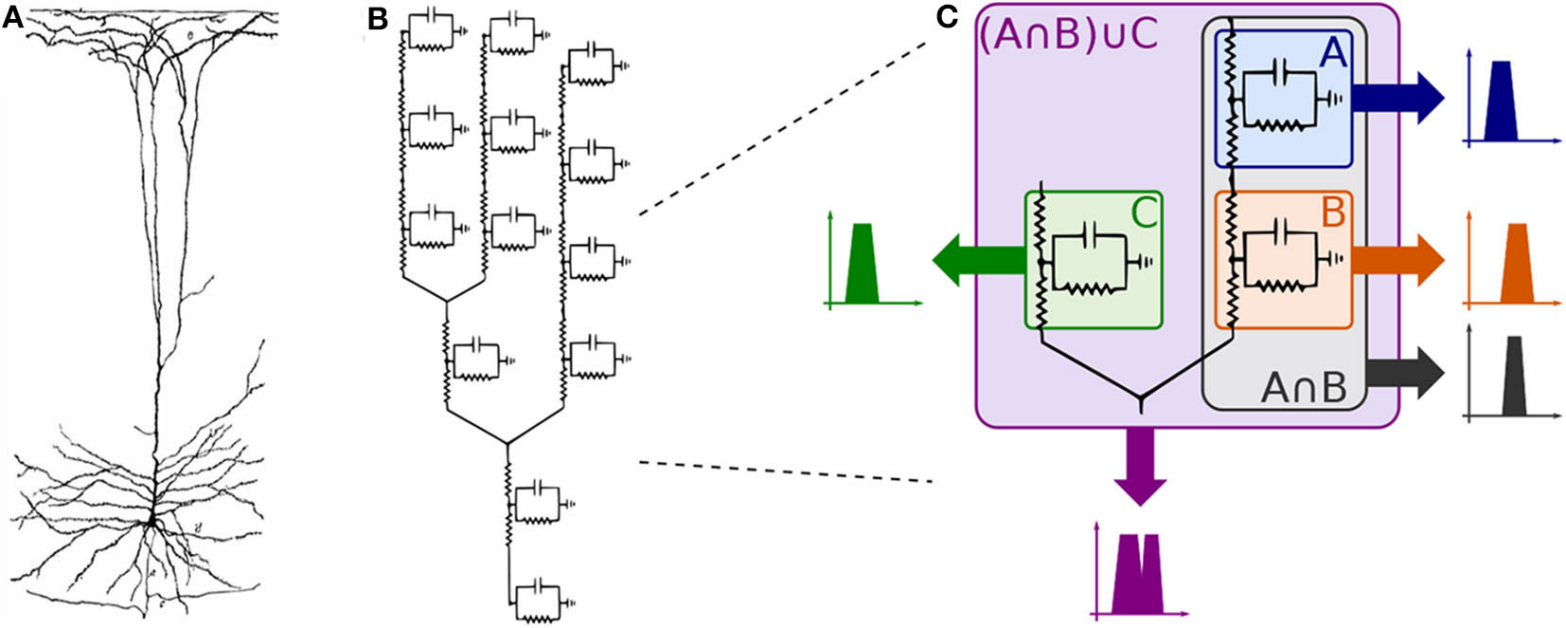
Compartmental neuron representation: **(A)** Natural topology of a pyramidal cell, **(B)** Electronic circuit compartments, and **(C)** Effects of serial and parallel connections between compartments.

Observing the neuron as a set of compartments described by transfer functions equivalent to that of (35), the neuronal morphology of a pyramidal cell, as illustrated in Figure 6A, (or any cell for that matter) can be modeled as an electrical circuit as shown in the topology of Figure 6B; the dendritic ramifications are modeled as a combination of serial and parallel connections terminating in the soma which is connected to the axon modeled as a series of compartments; its interpretation in terms of filtering is given in Figure 6C. The effect of a serial connection of two compartments is one of set-intersection when observed in the frequency domain: two bandpass filters in series pass only the frequencies that exist in both of their passbands. On the other hand, a parallel connection has a set-union effect, a parallel connection of filters will pass all the frequencies in both their passbands. As such, a large network (tree) of such compartments with similar bands combined in a cell, and cells combined in a group of cells will exhibit asymptotic bandpass behavior as well.

Every single compartment, each represented by one transfer function, is grouped in trees of three cells (Figure 1) forming a logic gate; the three gates are connected into a tree of their own, as illustrated in Figure 5A, forming a logic circuit. All of the cells are represented with the same form of the transfer function,

(36) $$\begin{array}{l} W_{i}\left(s\right)=\zeta_{i}\gamma_{i}^{-1}\frac{C_{i}^{-1}\gamma_{i}s}{s^{2}+C_{i}^{-1}\gamma_{i}s+\lambda_{i}^{-1}C_{i}^{-1}},\;i=1,\ldots ,9 \end{array}$$

with symbols defined previously, and a new parameter ζ_*i*_ describing the synaptic weight for the *i*th cell; ζ_*i*_ acts as a tunable gain for the neurons.

**Figure 7:**
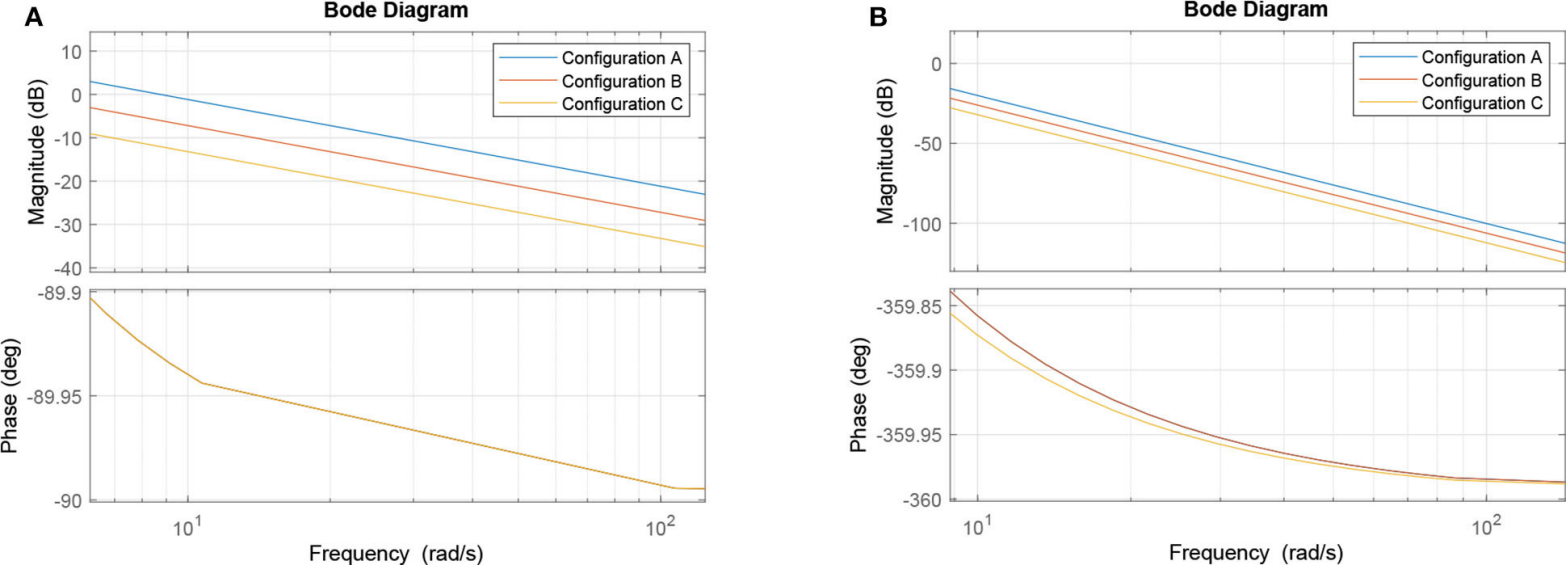
Bode plots: **(A)** Single second-order bandpass filter approximation and **(B)** Filter structure from Equation (37).

Using the parameters from (Mauro et al., 1970) aiming to keep them within the physically sensible orders of magnitude, we obtain the reference values of $\bar{\gamma }=0.0024$, $\bar{\lambda }=119$, $\bar{C}=1$ and $\bar{\zeta }=1$, and the values for 9 cells were generated multiplying these reference values by a uniformly distributed random variable in the range (0, 1). This kind of distribution is widely used to describe experiments where an arbitrary result should lie between certain boundaries, and in our case boundaries are defined by reasonable orders of magnitude around values made available by previous studies; keeping exactly the same parameters for all cells in the cascade is not realistic. The total transfer function of this system is

(37) $$\begin{array}{l} W=\left(\left(W_{1}+W_{2}\right)W_{3}W_{7}+\left(W_{4}+W_{5}\right)W_{6}W_{8}\right)W_{9}, \end{array}$$

and its frequency response (Bode plot) for the relevant range of frequencies in our applications (Wilson et al., 2004) is shown in Figure 7B.

Let us now observe three cases concerning the choice of ζ_*i*_ values. In the first case, we keep all of them at unity and consider it our base case for this part of the analysis (and to keep it aligned with the rest of the paper, we call it *Circuit B*). In the second case, we double the values of ζ_3_ and ζ_6_, which corresponds to the manipulation of the output cell for the two input gates in *Circuit A*. In our linear model, this is equivalent to doubling ζ_9_ and leaving everything else intact. Finally, in the third case, we manipulate the output cell of the last gate by halving its synaptic conductance (*Circuit C*). This effectively means that the three cases are ζ_9*B*_ = 1, ζ_9*A*_ = 2, and ζ_9*C*_ = 1/2, respectively. Since the tunable gain ζ_9_ of the gate *W*_9_, is the tunable gain of the whole system *W* according to (37), its change would offset the frequency response along the ordinate axis, i.e., lower gains (lower conductance) would suppress the unwanted frequencies in a better way, while higher gains would do the opposite. This is demonstrated in Figure 7A. The process of the analysis is summarized in Algorithm 1 and a summary with all elements from both the original and linearized versions of the Hodgkin-Huxley as well as the transfer function model is presented in Table 2.

**Algorithm 1:**
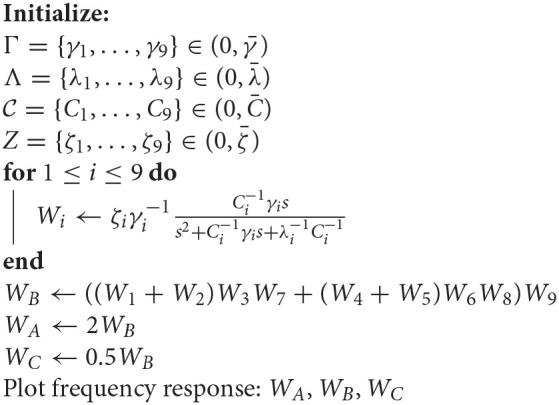
Linear model filter analysis

**Table 2:** Summary of elements described in the proposed model.

**Element**	**Description**
*C*	Membrane capacitance
*g*_*Na*_,*g*_*K*_,*g*_*l*_	Sodium, potassium, and leak conductances
*E*_*Na*_,*E*_*K*_,*E*_*l*_	Sodium, potassium, and leak reversal potentials
*I*_*ext*_	External stimulus
*I*_*Na*_,*I*_*K*_,*I*_*l*_	Ionic current for the sodium, potassium, and leak channels
*V*	Membrane potential
*m,h*	Sodium activation and inactivation variables
*n*	Potassium activation variable
α,β	Rate constants for *m*, *h*, and *n* from permissive to non-permissive state and vice-versa
δ	Small variation around the steady-state
*G*_*T*_	Total pure conductance
*G*_*Na*_,*G*_*K*_,*G*_*L*_	Sodium, potassium, and leak pure conductances
ḡ_*Na*_,ḡ_*K*_,ḡ_*l*_	Maximum attainable sodium, potassium and leak conductances
*m*_∞_,*h*_∞_,*n*_∞_	Steady-state values of *m*, *h*, and *n*
*g*_*m*_,*g*_*h*_,*g*_*n*_	Conductances of the inductive branches
*L*_*m*_,*L*_*h*_,*L*_*n*_	Inductances of the ionic paths
*W*	Transfer function of the filter
*K,Q*,ω_0_	Gain, selectivity, and peak frequency of the filter
ζ	Synaptic weight

Alternatively, as we suggested earlier, a single transfer function of a compartment serves as an approximation of the entire system due to the effects of repeated bandpass filtering in Figure 6C. In such case, we observe 20 dB/decade slope in the Bode plot shown in Figure 7A (as compared to 80 dB/decade slope in Figure 7B) and the same offset of 20 · log_10_2 ≈ 6 dB in case of halving/doubling the synaptic weight. Since the filter is of a band-passing nature, it is only natural that, around the resonant frequency, lower and higher frequency amplitudes should be ideally attenuated toward zero. Thus, it is worth mentioning that in both cases depicted here, the part of the frequency response with the cusp is at very low frequencies, so it is not visible in the relevant part of the spectrum. As such, the filter behaves as a low pass filter for all practical considerations.

## 3. Results

In this section, we discuss the simulation results concerning the reconfigurable logic gates as well as the circuits. For all simulations, intrinsic parameters of the cell were kept at their default values (such as the length and diameter of each of their compartments) meaning that nothing concerning their morphological properties was changed, the spike trains fed to the input of the circuits followed a *Poisson* process and the threshold for spike detection and data analysis was 0 mV where any potential higher than that in a specific time slot would be considered a bit “1,” characterizing the use of a simple *On-Off Keying (OOK)* modulation which was implemented where a spike is considered as a bit “1” and its absence a bit “0” in each time slot. The cell models and information on their respective connection probabilities between different pair of neurons were obtained from the work of Markram et al. (2015), and then we used NEURON and Python for simulation and data analysis (Carnevale and Hines, 2009; Hines et al., 2009). The source-code of our simulations is publicly available on a GitHub repository^1^.

### 3.1. Reconfigurable Logic Gates

In this work, we call “reconfigurable” logic gates, the gates that work by changing the synaptic weight between the connections of both input cells with the output cell in a neuronal logic gate structure. Aiming to measure individual gate accuracy, the spike trains in the inputs were randomly produced but we control their frequency variation, in other words, for each simulation, the frequency at all inputs was the same and any change in the frequency was performed for all inputs of the gates meaning that none of the simulations account for different frequency values between different inputs in a single simulation. The accuracy is a simple but powerful measure for the performance of the gates, with which we intend to analyze the effects of the dynamics of the cell on the output of the circuit when comparing this output with the ideal response of the circuit derived from its truth-table. The accuracy is calculated according to the following equation (Hanisch and Pierobon, 2017):

(38) $$\begin{array}{l} A\left(E\left[Y\right];Y\right)=\frac{P_{1,1}+P_{0,0}}{\underset{Y}{\sum }\underset{E\left[Y\right]}{\sum }P_{Y,E\left[Y\right]}}, \end{array}$$

where *P*_*Y,E*[*Y*]_ is the probability of *Y* given *E*[*Y*] in which *Y* is the actual output and *E*[*Y*] is the expected output and *Y&E*[*Y*] ∈ {0, 1}. *P*_*Y,E*[*Y*]_ resembles the conditional probabilities in a binary symmetric channel (BSC). Thus, *P*_0,0_ = 1 − *P*_1,0_, and *P*_0,1_ = 1 − *P*_1,1_. It is possible to calculate *P*_1,1_, for instance, by counting the number of bits there are for each input-output combination. In other words, considering *#B*_*i,j*_ the number of times a bit *i* was received when bit *j* was sent knowing that *i&j* ∈ {0, 1}, then *P*_1,1_ = *#B*_1,1_/(*#B*_1,1_ + *#B*_0,1_).

Given the objective of obtaining a behavior similar to an OR gate, the synaptic weight should be set to 0.06 μS, meaning that the pre-synaptic stimuli will drive a higher influence on the depolarization of the post-synaptic cell. On the other hand, for an AND behavior, the weight is set to 0.03 μS, which reduces the influence of a single spike and look to a response of the post-synaptic neuron only when two spikes arrive very close to each other in terms of time. This is conducted so we have acceptable levels of accuracy when compared to the expected outputs of the gate.

**Figure 8:**
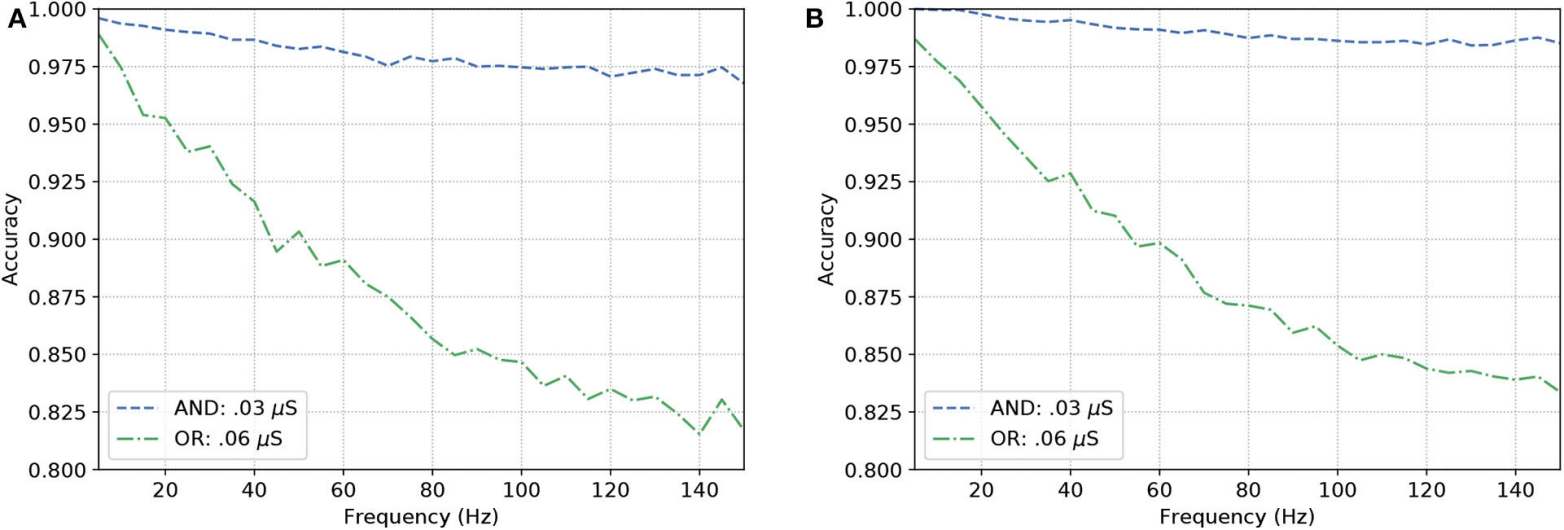
Analysis on reconfigurable logic gates with neurons of types **(A)** L23-MC, L23-NBC, and L1-DAC and **(B)** L23-MC, L23-NBC, and L1-HAC.

Figure 8 show similar responses when gates originally built to be of a specific kind. This means either OR or AND gates can change their configurations that drives their gating capabilities by modifying the synaptic weight between the connections of the input cells and the output cell. Although there is quite a visible difference between the performance of AND and OR gates, even at high frequencies (150 Hz), the accuracy of the reconfigurable logic gates remains above 80%.

### 3.2. Neuronal Logic Circuits

Once the reconfigurable behavior of the gates is assessed, they are connected to other gates to form a logic circuit. The performance is measured employing a ratio (frequency response), i.e., the number of spikes (bits “1”) in the output divided by the nominal input frequency, in Hertz. This ratio is also known as the magnitude, or gain when evaluating the data in decibels. Following the approach for individual gates, the inputs are random and the frequency is increased uniformly. Since the gates showed similar accuracy when increasing the input frequency, we picked the one analyzed in Figure 8A for our circuit analysis with a reconfigurable logic gate, modifying only the output gate's synaptic properties.

**Figure 9:**
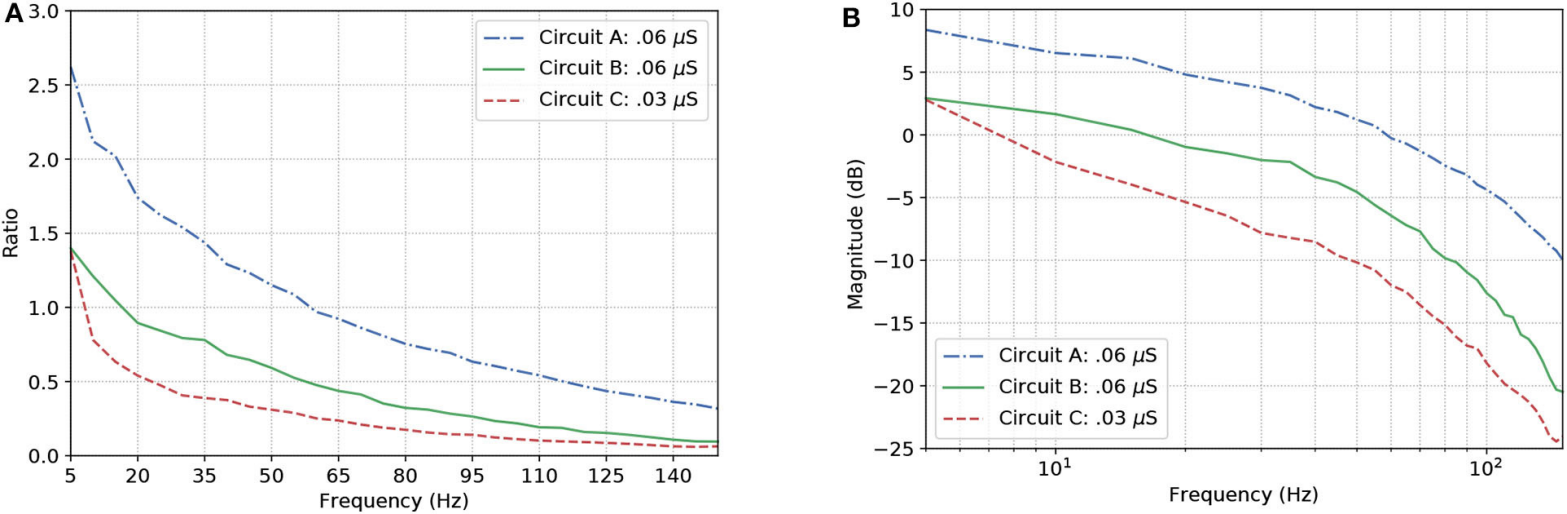
Effects of dynamic changes to the synaptic weight in circuits A, B, and C; **(A)** Frequency response and **(B)** Magnitude in decibels.

Figure 9A show the results for the circuits in Figure 5A. As expected, Circuit C has a stronger attenuation of the signals passing through it, and this is mainly due to the fact it is an arrangement with three AND gates and, based on the truth table, an AND gate only responds to stimuli if all its inputs are active at the same time. The magnitude in decibels shown in Figure 9B follow a standard presentation of the response of digital filters.

In the non-linear case of the system, the filtering is even better than what the linear model would promise, i.e., the suppression of unwanted frequencies is better due to superexponential decay. Let us compare Figures 7B, 9B. The linear model suggests that a constant difference of 6 dB is to be expected if the synaptic weight of the output cell is halved (or doubled), and a linear, constant amplitude drop. In the nonlinear model, we do observe a 20 dB/decade drop and 6 dB difference at relevant frequencies, but instead of a linear trend, we observe a convex response, which helps in attenuating high frequencies faster than we would expect from the linear model. This is because the linear model is accurate in a neighborhood of the point at which it was linearized.

Now, let us consider *H*(ν) as the response of an ideal low-pass filter, and *W*(ν) the response of the proposed neuronal filter, the counter-efficiency of *W* given *H* is calculated as

(39) $$\begin{array}{l} \psi \left(W|H\right)=\int_{0}^{\nu_{c}}|W\left(\nu \right)-H\left(\nu \right)|d\nu +\int_{\nu_{c}}^{\nu_{f}}|W\left(\nu \right)|d\nu \end{array}$$

where ν_*c*_ is the cut-off frequency and ν_*f*_ is the last evaluated frequency (in this relationship, the lower the value, the more efficient the filter is). Since, in terms of magnitude, a frequency band when cut by an ideal filter should be attenuated toward negative infinity (−∞), we have to pick a limit for the calculation of the area under the curves. In our case, after a visual inspection, the baseline for calculation chosen was −25 dB, because this is the closest integer value to the lowest values of magnitude.

**Figure 10:**
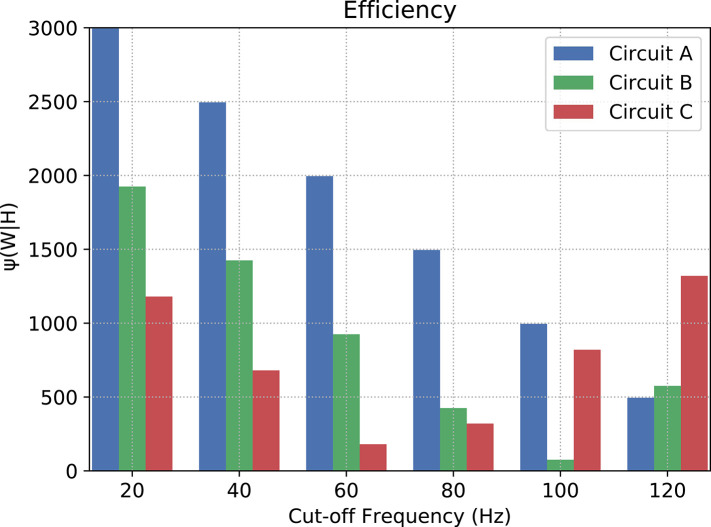
Counter-efficiency of the circuits when compared to ideal filters (the lower the value, the better the filter's performance).

Figure 10 depicts the counter-efficiency analysis performed for the three circuits. As it is shown, for different frequency bands we have some circuits performing better than others. Also, each circuit has a preferable frequency band for achieving maximum efficiency. For frequencies lower than or equal to 80 Hz, Circuit C seems the most efficient, especially at 60 Hz, while frequencies around 100 Hz show Circuit B as the most efficient which is also the band where it performs the best. Circuit A, on the other hand, has its best performance for 120 Hz, and probably for higher frequencies as well if the trend continues.

This shift in performance may allow us to control which type of circuit we want to activate inside the brain depending on which activity the subject is performing at the time, e.g., being awake or being asleep. These changes may be induced by the intake of specific drugs that alter synaptic properties in a neuronal connection.

**Figure 11:**
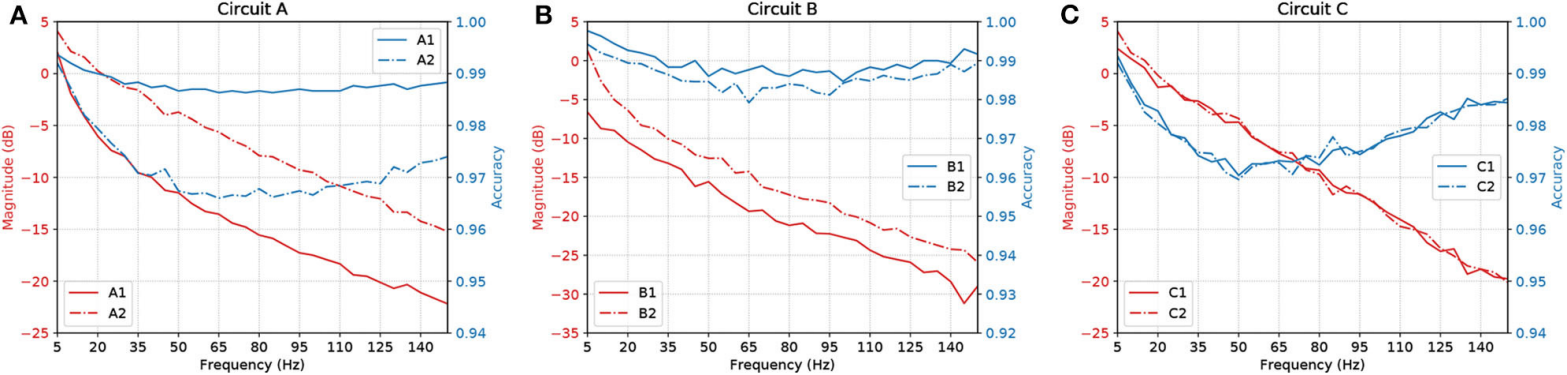
Parallel between Magnitude (dB) and accuracy of circuits **(A)** A, **(B)** B, and **(C)** C, with AND gates in cascade.

Figure 11 shows a parallel analysis between the magnitude in dB and the accuracy of the filters with AND gates in cascade. Each circuit is identified by a pair of characters, the first is the letter referring to the circuit analyzed, the second is how many AND gates were connected in cascade. For example, *A*2 means Circuit A with two AND gates in cascade, as illustrated in Figure 5B.

The results suggest that, by increasing the number of gates in cascade, we have to deal with attenuation in the network due to propagation caused by specific characteristics of the cell, such as the connection probability; hence, the more gates in cascade the worse the performance of the circuit. Also, even though the ratio keeps going downwards, at some point, the accuracy will start to shoot up. With careful evaluation, the dip in the accuracy along mid-range frequencies is very low in terms of scale, showing a difference of only around 0.03 on the values of accuracy.

## 4. Discussion

Synaptic weight plays a role in the influence of the pre-synaptic stimuli and its impact on the post-synaptic neuron and has a value proportional to the synaptic conductance (Gardner, 1989) which is driven by the amount and type of neurotransmitters that are being bound to the post-synaptic terminals. The higher the connection probability between pairs of neurons, the stronger the influence of a specific synaptic weight. This is due to the proportional relationship that the weight has with each synaptic connection that individually releases a certain amount of neurotransmitters, hence, different neuron types may affect the influence of a fixed value of synaptic weight. This explains how the accuracy values fluctuate between different types of gates and circuits as shown in Figure 11. Within a larger network spatial dimension, the types of neurons may drive a higher accuracy fluctuation since the network connection exhibits different synaptic weights between each other.

With our model, we have mainly investigated the attenuation on the spiking frequency for three different types of circuits in which we decrease the number of OR gates by replacing them with AND gates. We were also able to have the fine-tuning synaptic properties showing a difference of around 5 dB in performance between the curves in Figure 9B. Changes in the synapse are also considered (Vogels and Abbott, 2005), either by strengthening or weakening specific synaptic connections, logic gates were built within a homogeneous network of integrate-and-fire neurons. Moreover, the experiments conducted by Goldental et al. (2014) followed a procedure that enforced stimulations on neuronal circuits within a network of cortical cells *in-vitro* and they do propose other types of gates such as XOR and NOT. Furthermore, we increased the number of AND gates in a cascade-like manner in order to confirm that the longer the line of cascade gates, the more attenuated the signal should be if none of those elements receives any kind of external stimuli despite the spike coming from the circuit, and this result is depicted in Figure 11. A peak value in the difference of around 8 dB occurs in Circuit A, decreasing to around 5 dB in Circuit B and there is a small difference in Circuit C. The transfer function derived from the Hodgkin-Huxley linear model suggests a band-pass behavior of the system (Plesser and Geisel, 1999) for very low frequencies leaving us with a low-pass filter acting on higher frequencies ranging from 5 to 150 Hz. Considering the time for a spike to be fired that comprises depolarization, repolarization, and refractory period, higher frequencies will lead to saturation and non-realistic behavior of neuronal firing.

Our results, therefore, suggest that neuronal logic circuits can be used to construct also digital filters, filtering abnormal high-frequency activity which can have many sources including neurodegenerative diseases. A metric of counter-efficiency was also proposed, which should show how far apart the real results are from the ideal cases. We found that frequency bands were found to be of optimal value for different types of circuits such as 60 Hz for circuit C, 100 Hz for circuit B, and 120 Hz for circuit A, as shown in Figure 10. Based on the presented results, we demonstrate that by reconfiguring the gates inside the digital filters we can shift the intensity with how we attenuate the spiking frequency allowing an on-the-fly adaptation of the filtering tasks depending on the activity that is being performed by the subject where, for instance, circuit C should outperform both A and B for frequencies lower than or equal to 80 Hz.

The envisioned application of the proposed mathematical framework is for *in-silico* pharmacology and how it can be used to provide advanced prediction supporting computational strategies to test drugs. Since drug design and discovery in neuroscience are very challenging, especially due to the complexity of the brain and the significant impediment of the blood-brain barrier (BBB) imposes on the delivery of therapeutic agents to the brain. The success rate for approval by competent authorities of such drugs is <10%. Such a low rate is attributed not only to factors related to the disease itself, such as complexity, slow development, and gradual onset but also, to the limited availability of animal models with good predictive validity and the limited understanding of the biological side of the brain (Geerts et al., 2020). The system model derived from a set of coupled neuron compartments can help push forward the design of these neuronal filters and provide a platform for *in silico* drug-induced treatments on top of engineered biological models of neurons. A platform that could lead to cost-effective drug development and analysis of potential bio-computational units capable of enhancing signal processing in the brain, as well as predicting long-term effects of using a specific drug are potential uses of the proposed mathematical framework.

## 5. Conclusion

In this work, we proposed a reconfigurable spike filtering design using neuronal networks that behave as a digital logic circuit. This approach requires the cells to be sensitive to modifications through chemicals delivered through several proposed methods available in the literature. From the Hodgkin-Huxley action potential model we developed a mathematical framework to obtain the transfer function of the filter. This required a linearization of the Hodgkin-Huxley model that changes the cable theory simplification for each cell compartment. To evaluate the system, we have used our transfer function as well as the NEURON simulator to show how the frequency of operation, logic circuit configuration as well as logic circuit size can affect the accuracy and efficiency of the signal propagation. We observed that all-ANDs circuit produces more accurate results concerning their truth-table when compared to all-ORs. In addition, the results show that each digital logic circuit is also reconfigurable in terms of cut-off frequency of the filter, by manipulating the types of gates in the last layer of the circuit.

We believe the proposed filter design and its mathematical framework will contribute to synthetic biology approaches for neurodegenerative disorders such as epilepsy, by showing how the control of cellular communication inside a small population can affect the propagation of signals. For future work, we plan the use of non-neuronal cells, e.g. astrocytes, for the control of gating operations and the assessment of neuronal filtering capabilities at a network level. Treatment techniques based on this method can be a radical new approach to reaching precision and adaptable outcomes, inspired from electronic engineering as well as communication engineering. Such techniques could tackle at a single-cell level, neurons affected by seizure-induced high-frequency firing or bypass neurons that have been affected by a disease-induced neuronal death and degeneration, thus keeping the neuronal pathway working at a performance as optimal as possible.

## Data Availability Statement

The original contributions presented in the study are included in the article/supplementary material, further inquiries can be directed to the corresponding author/s.

## Author Contributions

GA performed the simulations and wrote the manuscript. HS performed the control-theoretic analysis. GA, HS, and MB performed the data analysis. SB, NM, MB, and MW led the work development. All authors contributed to manuscript writing and revision. All authors also have read and approved the submitted version.

## Conflict of Interest

The authors declare that the research was conducted in the absence of any commercial or financial relationships that could be construed as a potential conflict of interest.

## Footnotes

^1^https://github.com/gladonias/neuronal-filters
